# Supplementary material for: Connexin 43 Channels in Osteocytes Are Necessary for Bone Mass and Skeletal Muscle Function in Aged Male Mice
Source: Int J Mol Sci. 2022 Nov 4;23(21):13506. doi: 10.3390/ijms232113506 (PMC9654692; doi:10.3390/ijms232113506)
Supplement: Supplementary file 1 [file ijms-23-13506-s001.zip › ijms-1939906-supplementary.pdf]

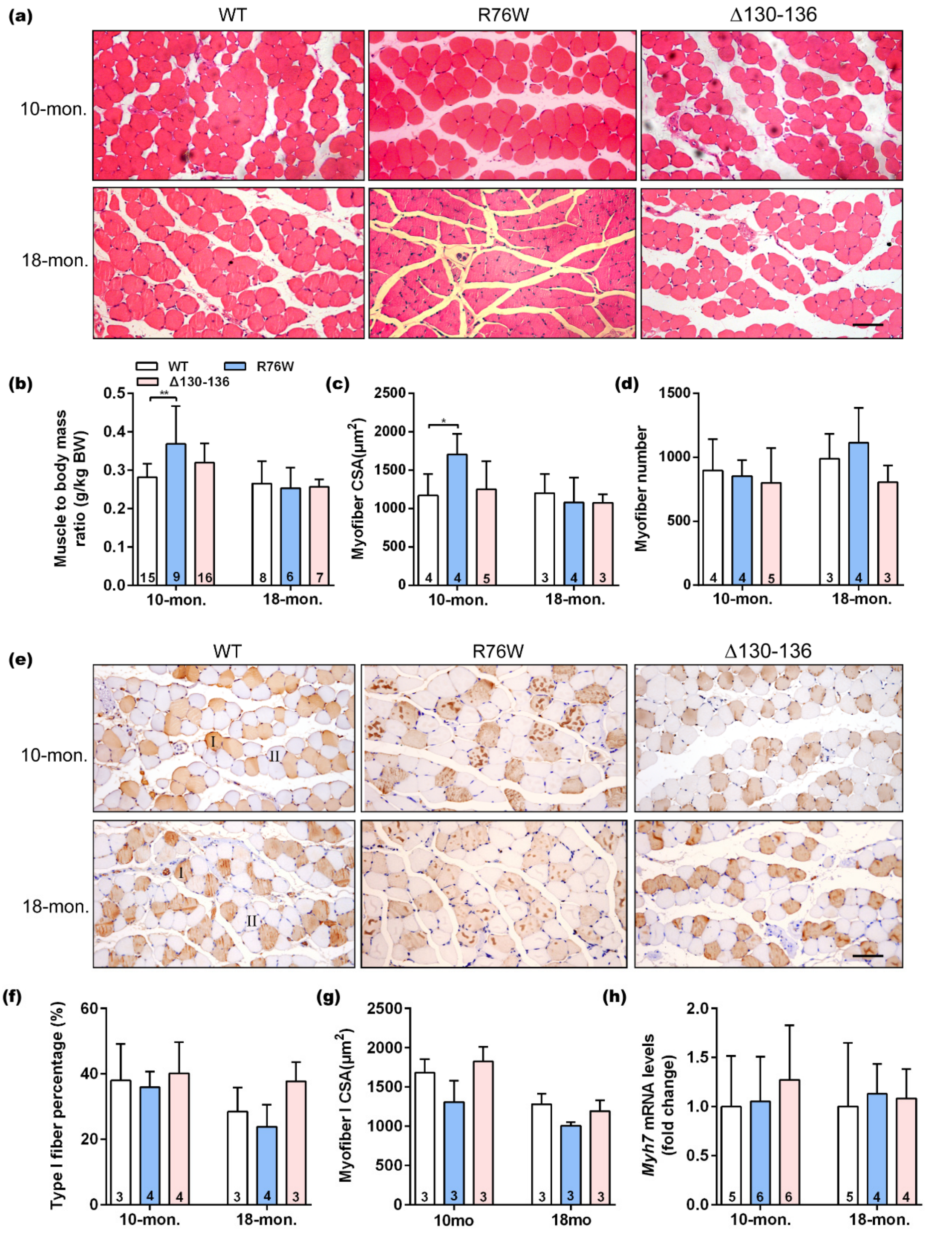

Figure S1. Effects of Cx43 channels on the muscle mass and myofiber size in 10- and 18-month-old transgenic mice. (a) Representative H&E staining images of myofibers in SL muscles of 10- and 18-month-old transgenic mice and their WT littermates. Scale bar=50μm. (b-d) Muscle mass (b), myofiber CSA (c) and myofiber number (d) in SL muscles were determined. (e) Representative immunohistochemical images of myosin heavy chain type I (MyHC I) myofibers in SL muscles. Scale bar=50μm (f-h) The proportion of MyHC I-stained myofibers in total myofibers (f), myofiber I CSA (g) and *Myh7* mRNA expression (h) in SL muscles. $^{*}P<0.05$; $^{**}P<0.01$.

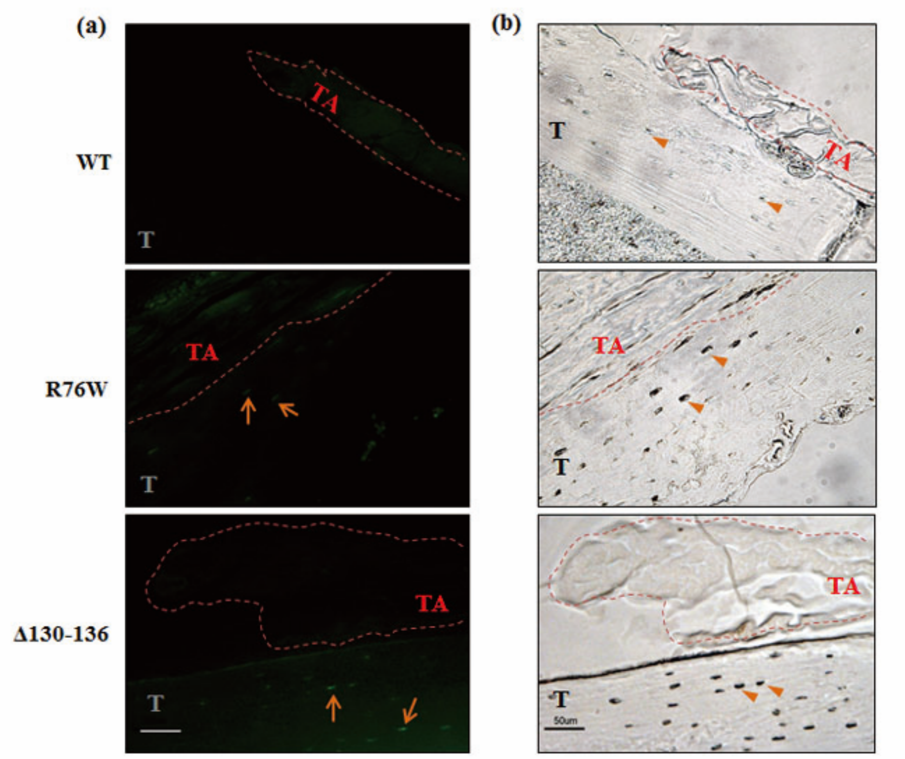

Figure S2. Histological evaluation of 10-kb Dmp1-GFP expression in transgenic mice. GFP expression (orange arrows) was mainly present in the osteocytes (orange arrowheads) within cortical bone of tibia (T) but not in the nearby tibia anterior (TA) muscle. (a) Representative fluorescence image and (b) phase contrast image taken from the same area. The dotted light red frames represent the region of TA. Bar=50μm.

Table S1. Primer sequences for RT-qPCR

Target genes	Primer sequences(5' to 3')	Annealing temperature(℃)
mCx43	Forward: CGGAAGCACCATCTCCAACT<br>Reverse: CCACGATAGCTAAGGGCTGG	60℃
mCx50	Forward: CAAGGGCTGTCTGCTGAGAA<br>Reverse: AGATCATCTGACCTGGCCCT	60℃
Alp	Forward: GTTGCCAAGCTGGGAAGAACAC<br>Reverse: CCCACCCCGCTATTCCAAAC	60℃
Myh7	Forward: CAACCTGTCCAAGTTCCGCA<br>Reverse: TACTCCTCATTCAGGCCCTTG	60℃
NFATc-1	Forward: GACCCGGAGTTCGACTTCG<br>Reverse: TGACACTAGGGGACACATAACTG	60℃
GAPDH	Forward: TCAACAGCAACTCCCACTCTTCCA<br>Reverse: ACCCTGTTGCTGTAGCCGTATTCA	60℃
